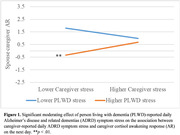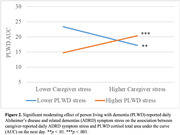# Daily Perceptions of Dementia Symptoms Within Care Dyads: Links to Diurnal Cortisol Rhythms in Couples Living With Early‐Stage Dementia

**DOI:** 10.1002/alz.095633

**Published:** 2025-01-09

**Authors:** Courtney A. Polenick, Angela Turkelson, Charity Garner, Nikita R Daniel, Shreya M Salwi, Diarratou Kaba, Kira S. Birditt

**Affiliations:** ^1^ University of Michigan, Ann Arbor, MI USA

## Abstract

**Background:**

Symptoms of Alzheimer’s disease and related dementias (ADRD), including memory problems, are often stressful for people living with dementia (PLWD) and family caregivers. However, we know little about daily perceptions of ADRD symptoms and links to physiological stress reactivity within care dyads. We evaluated how daily ADRD symptom stress (i.e., perceived stress related to ADRD symptoms) reported by PLWD and their spouse caregivers predict diurnal cortisol rhythms among heterosexual couples living with early‐stage ADRD.

**Method:**

Participants included 39 PLWD (*M* = 72.1 years, *SD* = 7.6, range = 52‐86 years) and their spouse caregivers (*M* = 70.7 years, *SD* = 9.0, range = 49‐88 years) who both completed 7 consecutive days of brief morning and evening phone interviews and collected saliva samples (four times per day) during four of those days. Outcomes included the cortisol awakening response (AR; awakening to 30 min post‐awakening), decline (DEC; 30‐min post‐awakening to bedtime cortisol), and total area under the curve (AUC). Multilevel models controlled for study day, age, gender, and education.

**Result:**

Higher caregiver‐reported daily ADRD symptom stress was associated with their own higher AR on the next day when PLWD‐reported daily ADRD symptom stress was high (*b* = 1.04, *SE* = 0.38, *p* = .006). Higher caregiver‐reported daily ADRD symptom stress was associated with a higher AUC among caregivers (*b* = 2.51, *SE* = 0.87, *p* = .006) but a lower AUC among PLWD (*b* = ‐1.65, *SE* = 0.69, *p* = .021) on the same day. Higher caregiver‐reported daily ADRD symptom stress was associated with a lower PLWD AUC on the next day when PLWD‐reported daily ADRD symptom stress was low (*b* = ‐5.75, *SE* = 1.90, *p* = .002) but a higher PLWD AUC when PLWD‐reported daily ADRD symptom stress was high (*b* = 5.27, *SE* = 1.51, *p* < .001). Caregiver‐reported and PLWD‐reported daily ADRD symptom stress were not associated with DEC.

**Conclusion:**

Daily ADRD symptom stress reported by spouse caregivers was associated with physiological stress reactivity among caregivers and PLWD. These findings inform targets of clinical care and interventions to improve the well‐being of both care dyad members.